# Factor Structure of the “Top Ten” Positive Emotions of Barbara Fredrickson

**DOI:** 10.3389/fpsyg.2021.641804

**Published:** 2021-05-14

**Authors:** Leopold Helmut Otto Roth, Anton-Rupert Laireiter

**Affiliations:** ^1^Faculty of Psychology, Institute for Clinical and Health Psychology, University of Vienna, Vienna, Austria; ^2^Department of Psychology, Division of Psychotherapy and Gerontopsychology, University of Salzburg, Salzburg, Austria

**Keywords:** positive psychology, positive emotions, exploratory and confirmatory factor analysis, structural analysis, model comparison

## Abstract

In order to contribute to the consolidation in the field of *Positive Psychology*, we reinvestigated the factor structure of top 10 positive emotions of Barbara Fredrickson. Former research in experimental settings resulted in a three-cluster solution, which we tested with *exploratory* and *confirmatory* methodology against different factor models. Within our non-experimental data (*N* = 312), statistical evidence is presented, advocating for a single factor model of the 10 positive emotions. Different possible reasons for the deviating results are discussed, as well as the theoretical significance to various subfields in *Positive Psychology* (e.g., therapeutical interventions). Furthermore, the special role of *awe* within the study and its implications for further research in the field are discussed.

## Introduction

Through the rise of Positive Psychology in recent years, the role of *positive emotions* developed to become a core field of interest in *Positive Psychology*. This trend was largely based on the efforts of Fredrickson (1998), who became well-known in the field since her widely discussed article in the *Review of General Psychology* on *positive emotions* was published. Furthermore, in 2013, she declared her definition of the 10 most important positive emotions, based on her scientific experiences and findings in the last 2 decades (Fredrickson, 2013). These emotions, namely *joy*, *gratitude*, *serenity*, *interest*, *hope*, *pride*, *amusement*, *inspiration*, *awe*, and *love*, overlap partially with already existing measures from behavioral science, however, specific attribution of relevance was sparse. While the defined emotions have been referenced widely and have been picked up in mainstream media as well, structured research on factors in positive emotions had not received broader attention yet. Most studies, investigating factor structures in emotions or affect usually focus on existing questionnaires and scales. In terms of affect, which is, following Russell (2003), a more accessible, “raw” and ongoing evaluation of the personal state, factor replications of the widely used *Positive and Negative Affect Schedule* (PANAS, Watson et al., 1988) by Allan et al. (2015) or Seib-Pfeifer et al. (2017); Fredrickson (2013) might have been the most frequent and fruitful endeavors in the field of emotion research. The interrelated yet distinct concept of emotions (Izard, 1977) holds more room for differentiation, such as questions for state and trait or multiple levels of perception and cognition, involved in the process. Fredrickson (2013) offered comments on (a) the specific appraisal theme, (b) related thought-action tendency, and (c) accrued resources. These will be revisited partly in the below offered description of emotions and supplemented with more recent findings. So far, the bandwidth of measures and lack of consensus on a best practice scale hinders systematic research and structured overviews. Some articles indeed shed light on factor structures, such as Güsewell and Ruch (2012), taking empirical perspectives on the *Dispositional Positive Emotions Scales* (Shiota et al., 2006) or earlier by Argyle and Crossland (1987), however, classically, positive emotions remained either in the role of the dependent or independent variable, given the respective hypothesis and rarely became the subject of latent structure analysis.

### The Top 10 Positive Emotions

Considering the framework by Fredrickson (2013), the relevant emotions are described by her in their individual nature. She also considers their frequency in human experience, starting with *love* and *joy* and ending with *awe*. To her understanding, *love* is to be seen as special case, when describing emotions as she defined the state as it is of certain complexity in terms of appraisal, action tendencies, and personal resources. Cacioppo (2018) offers a more recent summary on the neurological findings of *love* in the context of brain circuits, networks, and the history of neuropsychological investigations.

*Joy* as high-frequency and relevant emotion is described by Fredrickson (2013), considering the definition by Frijda (1986), as *free activation*. As stated by Watkins et al. (2018), the understanding of *joy* and its role in Positive Psychology was too simplistic for a long time and the experience of the emotion holds more complex facets, such as spiritual longing or a sense of positivity in the face of struggle or difficulties, which yet have to be investigated.

Described as rather frequent as well, Fredrickson (2013) described *gratitude* as relevant emotion, also intertwined with *joy* as also reported by Watkins et al. (2018). The beneficial role of *gratitude* in the sense of clinical and non-clinical practice is extensively discussed in the literature and summarized in meta-analytic studies (Ma et al., 2017; Cregg and Cheavens, 2021).

The appraisal of *serenity* is described as *safe or familiar* by Fredrickson (2013) and to trigger tendencies such as *savoring* and *integrating*. It is further meant to help reflecting on one’s own priorities and circumstances. Disentangling the role of the given emotion, Soysa et al. (2021) described the predictive value of different facets of *serenity* on well-being, beyond their measures of mindfulness and therefore supporting a specific relevance of *serenity* in Positive Psychology. A different predictive approach was reported by Naz et al. (2020) by reflecting on the role of mindfulness, spirituality, and serenity in elderly persons.

*Interest* as emotion was reported as combination of *safety* and *novelty*, which offers the frame for learning and exploration. Current emotion-studies, focusing specifically on the complex of *interest* in psychology are rare, yet Su (2020) offers an overview of relevant *interest* research as well as an integrated model for understanding *interest* in its complexity.

According to Fredrickson (2013), *hope* plays a special role within the positive emotions, as it is the only one, not in the general context of safety, referencing the definition by Lazarus (1991), but arises on the interplay with fear. Further, the emotion is interrelated with optimism and resilience as also supported meta-analytically and in primary research (Alarcon et al., 2013; Munoz et al., 2017; Yıldırım and Arslan, 2020).

The concept of *pride* is framed as force of motivation, facilitated by given achievements. In a therapeutic context, Cohen and Huppert (2018) showed the significant role of *pride* in social anxiety as the central aspect for generally lowered positive affect and therefore a relevant position to consider in clinical settings. Providing further insight on the conceptualization, Dickens and Robins (2020) provide a meta-analysis on the dichotomized framing of the emotion in authentic and hubristic *pride*, showing reversed effects and supporting the beneficial role of authentic *pride* in mental health.

Less serious yet important, *amusement* is contextualized with laughter, social incongruity, and social bonds (Gervais and Wilson, 2005; Fredrickson, 2013). The role of humor in the social context was frequently studied and example wise summarized by Hall (2017), reporting consistent positive effects of positive humor styles on relationship satisfaction in over 43 samples.

The second least frequent emotion in concept of Frederickson (2013) is *inspiration*, described as object-centered interpersonal experience in the light of observing others performance. In terms of modern applicability, Meier and Schäfer (2018) showed uplifting effects of social comparison on *inspiration*, mediated by benign envy, measured in the context of social media, which was empirically extended in the following (Meier et al., 2020).

Last, Fredrickson (2013) lists *awe* as one of the positive emotions and as the least frequent one. This seems appropriate, as it is conceptualized as the perception of something, bigger than life and following changes in worldviews (Shiota et al., 2007). The emotion seems to hold a complex structure, as Yaden et al. (2019) reported a six-factor structure of the concept, including aspects such as *need for accommodation*, *self-diminishment*, or *perceived vastness*. As the body of literature on more diverse concepts and perceptions of *awe* is growing, its role will be discussed later in the text.

### The Current Study

Given the rising awareness in scientific literature regarding the role of positive emotions in daily life (Seligman and Csikszentmihalyi, 2000; Cohn et al., 2009), work environment (Diener et al., 2020), and health behavior (Seaton et al., 2018; Van Cappellen et al., 2018; Nylocks et al., 2019) a critical understanding of their possible structure and interplay seems a valid goal for academic perspectives. Further, recent findings on the therapeutic value of Positive Psychology and positive emotions is adding further relevance toward an improved knowledge on the underlying dynamics (Seligman et al., 2006; Guo et al., 2017; Ochoa et al., 2017; Mohamadi et al., 2019; Tagalidou et al., 2019; Furchtlehner et al., 2020) for clinical application.

To our knowledge, Hu et al. (2017) were the first research group to examine perspectives on defined emotions of Fredrickson (2013) by investigating their interplay. Using EEG-measurement (*N* = 20) and experimental emotion elicitation through videos, a three-cluster-system of the 10 emotions has been found, structuring the emotions in *encouragement* (*awe*, *gratitude*, *hope*, *inspiration*, and *pride*), *playfulness* (*amusement*, *joy*, and *interest*), and *harmony* (*love* and *serenity*). These findings have been replicated by Hu et al. (2019; *N* = 13) using *fNIRS* for measuring hemodynamic responses to emotional experience and a similar experimental procedure as in the first study.

In our study, we aim to extent the perspective on the top 10 emotions, using a naturalistic setting and larger sample size, where participants rated their subjective emotion experience within the past 2 days. Our landmark in the study was the question whether we would find the proposed clusters also represented as latent factors in our data and if the structure would generalize outside the laboratory and with everyday experiences of emotions and therefore without eliciting stimuli. This was done, using exploratory and confirmatory factor analysis and the comparison of empirically derived models.

## Materials and Methods

### Sample

We gathered information of 312 participants through online questionnaires. 62.2% identified as female, 37.2% as male, and 0.6% as diverse. The age ranged from 18 to 79 years (*M* = 29.11, *SD* = 10.10). Further demographic questions were assessed (*see measures*) and are summarized in a table in the Supplementary Material.

### Measures

Participants’ top 10 positive emotions (e.g., “gratitude”/“Dankbarkeit”) were assessed on a five-Point Likert Scale (1 = *not at all*, 5 = *very strongly*), which inquired as to the intensity with which participants had experienced the respective emotions within the last 48 h. The used German translations for Fredrickson’s emotions can be found in the Supplementary Material. Furthermore, we asked participants for their education, their subjective and objective income, best identified relationship status, mother tongue, parents’ mother tongue, best identified religion, housing situation and whether they followed an active sex life. A summary of the answers in percentages can be found in the Supplementary Material for structural comparison in following studies.

### Procedure

In fall 2018, the online study was spread through social media. The study was described as being conducted in German and no reimbursement for participation was advertised. The duration for completion was estimated for roughly 10 min. Participants were also asked to complete further scales, originated from *Positive Psychology*, for different purposes. To avoid sequential effects, the emotion questionnaire was placed in the beginning of the survey, after participants gave their informed consent to participation and confirmed to be above the age of 18. More details can be found in the Supplementary Material (further scales, used platforms, data handling, and treatment of missing values).

### Data Analysis

All computations have been conducted in *R 3.6.3* (R Core Team, 2020) and *RStudio 1.1.453* (RStudio Team, 2020). Exploratory data analysis was partially conducted in *JASP* (JASP Team, 2020). The data was initially opened in Excel for Mac.

To evaluate our data statistically, we applied descriptive analysis to our demographic measures, as well as the rated emotions. To check for consistent reporting, the general test for granularity-related inconsistency of means (*GRIM*) by Brown and Heathers (2017) has been applied to the means of emotions. Further, reliability analysis, computing Cronbach’s alpha was used on the 10 emotions.

As a first step toward latent structure analysis, we computed *pearson’s correlations* to observe inter-emotion dynamics as well as checks for eligibility of the data for *exploratory factor analysis* with *Kaiser-Meyer-Olkin Test* and *Bartlett test for sphericity*.

The *exploratory factor-analytical procedure* included the computation of Eigenvalues, visual scree plot inspection. Deriving from these findings and the cluster-approximation by Hu et al. (2017, 2019), we constructed alternative models for *confirmatory factor analysis*, varying the number of latent factors, included emotions and factor-correlation. This resulted in the comparison of eight models, using traditional model-fit indices (*RMSEA*, *CFI*, and *TLI*) as well as *BIC* and *AIC*, which have been used as selection criteria between the alternatives.

## Results

### Descriptive Statistics and Reliability

**Figure 1:**
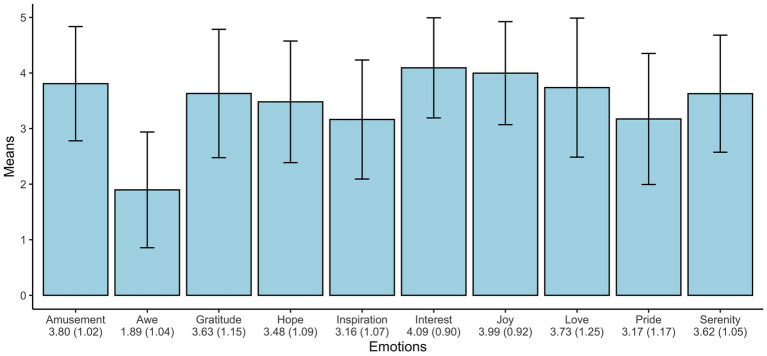
Means and SDs (in parentheses) of the “Positive Ten”.

The comparison of the emotions within the sample unveils a relatively consistent picture, except for *awe*. Figure 1 displays the observed means (*N* = 312) for all of top 10 positive emotions of Fredrickson (2013). The *Grim* test supported consistency of the reported values.

Furthermore, a reliability analysis, including and excluding *awe* was conducted as well and showed a marginally better reliability when *awe* was excluded [Cronbach’s ɑ: with *awe* 0.84, 95% *CI* (0.81;0.86); without *awe* 0.85, 95% *CI* (0.82;0.87)]. However, this observation could not be interpreted as meaningful, due to the small difference.

### Correlation of Emotions

Using Pearson’s correlation between all measured emotions, we observed a wide range of significant relationships between almost all variables. After already observing a generally low expression of experienced *awe* in our sample, this emotion also showed to be least connected to the other emotions, in terms of effect sizes. Apart from *awe*, the remaining emotions are all significantly correlated with each other (see Table 1).

**Table 1:** Correlations of measured emotions.

	1	2	3	4	5	6	7	8	9	10
Amusement	1	0.095*ns*	0.444	0.297	0.425	0.688	0.395	0.406	0.593	0.367
Awe		1	0.203	0.136	0.216	0.226	0.115	0.119	0.138	0.165
Gratitude			1	0.522	0.332	0.506	0.349	0.401	0.434	0.340
Hope				1	0.316	0.363	0.269	0.311	0.256	0.311
Interest					1	0.593	0.258	0.317	0.409	0.380
Joy						1	0.423	0.462	0.644	0.392
Love							1	0.355	0.286	0.178
Pride								1	0.414	0.395
Serenity									1	0.410
Inspiration										1

ns, not significant, all other correlations showed significant correlations (*p* < 0.05), exact *p*-values can be found in the Supplementary Material.

### Eligibility for Factor Analysis and Eigenvalue Inspection

With respect to the *Kaiser-Meyer-Olkin criteria* for sampling adequacy, an overall *MSA* of above 0.50 is the minimum requirement to complete a rational factor analysis (Kaiser, 1974). Our data reached a *MSA* = 0.88, which qualifies the data for further analysis. Also, the *Barteltt test for sphericity* reached significance (*p* < 0.0001). Additionally, we conducted a parallel analysis to determine the number of factors within the 10 emotions. The results clearly advocated for a one-factor solution. The observed data showed one Eigenvalue of 3.75, with a second largest value of 0.33. Also, visual inspection of the screen plot indicated one factor. The list of Eigenvalues can be found in the Supplementary Material.

### Confirmatory Factor Analysis and Model Comparison

**Figure 2:**
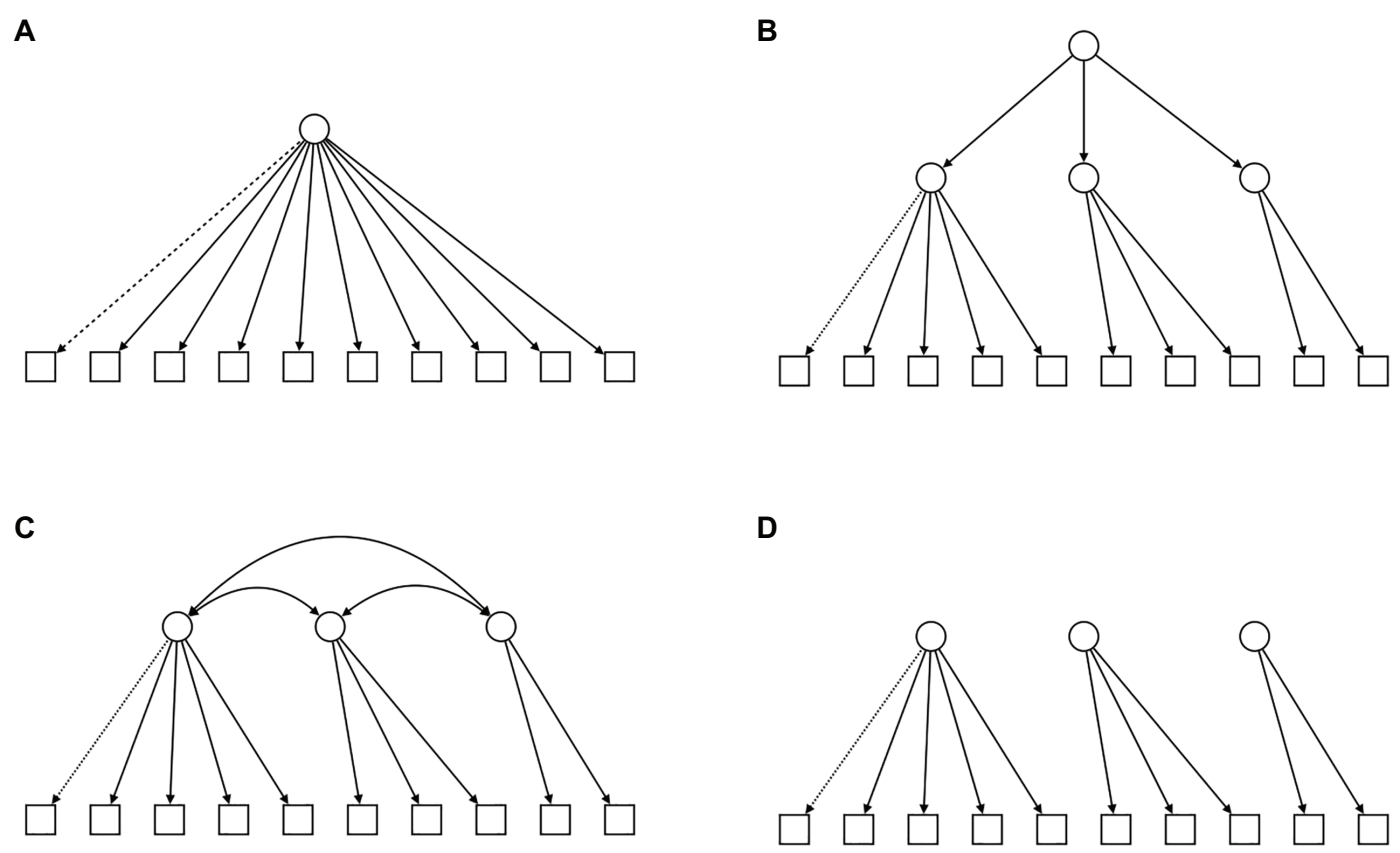
The figure summarizes the four core models, compared by confirmatory factor analysis. The dashed line references the challenging role of awe and refers to that we computed every model twice (with/without awe). **(A)** Shows the simple one-factor model, as suggested in our exploratory parallel analysis, **(B)** illustrates a hierarchical model with a higher-order general factor, above the proposed three first-order factors, **(C)** shows the intercorrelated three-factor model, and **(D)** the orthogonal alternative without factor intercorrelations.

To come to an adequate model fit we compared several options. We started by computing the suggested three-cluster solution from the findings of Hu et al. (2017, 2019), modeling the three factors (*encouragement*, *playfulness*, and *harmony*) and their including emotions and allowed inter-factor correlation (model C in Figure 2). Secondly, we tested the one-factor solution, empirically derived from our exploratory parallel analysis (model A in Figure 2). After observing strong factor correlation (0.79, 0.98, and 1.10), we also computed thirdly an orthogonal model (D in Figure 2) restricting the factors from interacting, as they showed nearly complete correlation. Lastly, we tested a hierarchical model, including an overall second-order factor (model B in Figure 2). In addition, as *awe* showed much lower factor loadings compared to the remaining nine emotions, we computed every model without *awe* as well.

To compare the eight models (A–D, with and without *awe*) and determine their match with the collected data, we ran *confirmatory factor analysis*, using the *lavaan* package (Rosseel, 2012) in *R*. As we compared non-nested models, we focused on *BIC* and *AIC* as criteria for model comparison, however, we integrated other typical fit indices in our report (*CFI*, *TLI*, and *RMSEA*; Table 2). *BIC* and *AIC* imply better model fit, if their value is smaller. As *lavaan* issued warnings regarding the factor correlation above 1.0 in the non-orthogonal three-factor model, we do not report its fit values. During computation, the hierarchical models showed problems with negative variances, mainly nested within the first-order factor harmony. Inspecting the *modification indices*, we discovered frequent recommendations of direct paths between discrete emotions and the general second-order factor representing a strong argument for us for a single factor solution. We therefore italized the indices for the hierarchical models, as their solution did not appear sufficiently trustworthy to us.

**Table 2:** Fit indices of compared models, sorted by AIC from highest to lowest.

	AIC	BIC	CFI	TLI	RMSEA
Model D with awe	8,677	8,752	0.575	0.454	0.199
Model A with awe	8,328	8,403	0.917	0.893	0.088
*Model B with awe*	*8,289*	*8,375*	*0.958*	*0.941*	*0.065*
Model D without awe	7,779	7,847	0.572	0.430	0.226
Model A without awe	7,431	7,498	0.919	0.892	0.098
*Model B without awe*	*7,393*	*7,472*	*0.960*	*0.940*	*0.073*

Italization indicates issues in the covariance matrix, the results should not be interpreted.

### Further Inspection of One-Factor Model Without Awe

Given the most robust combination of interpretability, parsimony, and strongest performance in information criteria, model A with nine remaining variables showed acceptable model fit, compared to the orthogonal three-factor solution, but worse model fit, compared to the non-interpretable hierarchical solution. The by-far strongest modification index suggested including covariances between *gratitude* and *hope*, and second strongest included covariances between *interest* and *joy*. Both modifications improved model fit in every aspect (Table 3).

**Table 3:** Effect of model optimization on fit indices.

	AIC	BIC	CFI	TLI	RMSEA
Original model	7,431	7,498	0.919	0.892	0.098
With first modification	7,391	7,462	0.960	0.944	0.071
With both modifications	7,382	7,457	0.970	0.956	0.062

In sum, we strongly recommend the single-factor model without *awe*, which takes inter-emotion covariances to some extent into consideration. However, the model also performed best with respect to information criteria without optimization, in contrast to the alternative models (Table 3).

## Discussion

### Summary

Inspecting our data, we found three distinct results. First, we found strong hints for a single factor solution of the observed emotions, empirically derived from *exploratory* and *confirmatory* factor analysis. Second, we identified *awe* as a questionable member of the top 10 positive emotions, as its experienced frequency did not follow that of the other emotions and model fit increased by excluding awe from analysis. Third, we found relevant model fit improvements by taking inter-emotion covariation into account, which appeared to us as support for further exploration of the interplay between emotions and a more diverse picture on single emotions.

### The Special Role of Awe

Regarding the role of *awe*, Hu et al. (2017, 2019) reported a more diverse picture of correlations with other emotions, ranging from 0.05 to 0.60, but also reported negative correlations, as for example, with *joy* (−0.17). In both, studies of Hu et al. and ours, the general expression of *awe* was relatively low compared to the other emotions, which present a more homogenous picture. There is a variety of possible explanations for this phenomenon. First, sample differences and artifacts could have biased the expression of *awe*, what should be investigated in detail in future studies. Second, cultural or linguistic differences, not yet evaluated, could determine different perspectives and therefore different expressions of *awe*. Third, and maybe most likely, *awe* might not be a classical *daily life emotion*, being normally experienced, compared to the emotion induction by Hu et al. (2017, 2019). Yet this can be limited to cultural and social restraints, when comparing the studies.

Hence, while Fredrickson (2013) derived the top 10 emotions rather theoretically, it seems in question as to whether *awe* should actually be part of the empirically based most important positive emotions, as the empirical evidence for its relevance appears unclear. Yet, it could also be seen as less frequent, but maybe occasionally relevant, which was not captured by our design, but would go in line with perspective on emotion frequencies of Fredrikson (2013).

As seen in the stimuli of Chirico et al. (2017) as well as in other *awe*-focused studies, researchers incorporate quite “high intensity” stimuli to elicit this emotion, such as “vastness” or “being moved” (Pelowski et al., 2019). With the same argument in the background, Shiota et al. (2007) suggested to elicit *awe* with stimuli such as music, art, or nature (in this case, massive natural experiences), due to its focus on cognitive functions. Yet, Chirico et al. (2017) using *virtual reality* simulations demonstrated the immersive potential of technology in eliciting this emotion. This focus on complexity is also demonstrated in a linguistic analysis conducted by Darbor et al. (2016) who centered their research on the pattern of incorporated words. While the authors found less vocabulary classically related to positive emotions, when compared to happiness, the focus of participants was driven toward complexity and challenges in perception of reality. Further, Nelson-Coffey et al. (2019) offer challenges to the positive conceptualization of awe in the light of studies, supporting a more positive relevance for self-transcendence.

Keeping these approaches toward *awe* in experimental psychology in mind, one may remember her or his last 48 h and wonder how often massive natural entities and challenges to her or his own perception of reality have occurred, which can therefore be seen as a presumable explanation why *awe* showed different patterns in the participants and why it needs artificial stimuli to be observed more clearly.

Additionally and equally important too, it seems not entirely clear to what extent *awe* is an entirely positive emotion as it is often conceptualized as containing a threat- or fear-related component (Takano and Nomura, 2020). And in one study, Guan et al. (2019) even distinguish between positive and threat related *awe*. Both groups found common brain patterns for both types of *awe*, however, presented distinct activation schemas. By the way, however, the mere opportunity to find one of the top 10 *positive emotions* as partially threatening raises questions to its belonging to the class of positive emotions. We would therefore propose examining the possibility of developing a third set of emotions with ambivalent valence, which can be dependent on the particular setting in which it arises, for example, as suggested with respect to *surprise* by Fredrickson and Losada (2005).

### Significance of Latent Structures in Positive Emotions

Also, with respect to the recently emerging field of interventions and therapeutic approaches with a focus on Positive Psychology (Furchtlehner et al., 2020), it seems worthy to investigate, how positive emotions are structured and related. This knowledge can guide developing tailored treatments, depending on whether specific clusters or a general factor in positive experience of emotions is affected. This also raises questions as to the underlying basic dynamics between emotions and whether inter-emotional compensability is possible. Furthermore, it would be interesting to examine to which extent the experience of a broad scope of positive emotions appears relevant to general well-being and other psychological measures (Tagalidou et al., 2019). As we observed relevant increase in the model fit by allowing covariation of emotions, more in-depth understanding of these could be helpful for future advances. So far, general emotion scales pay little attention to the “*what*” in measuring emotions, as they usually contribute to an average of experienced emotions for further analysis. On the other hand, some studies closely investigate single emotions in specific questionnaires, however, no holistic picture of possible other influencing variables is drawn. Our findings on the combination of *gratitude* and *hope* as well as *interest* and *joy* are partially supported by earlier findings. Loo et al. (2014) found *gratitude* and *hope* as protective factors in problematic gambling behavior and Proyer et al. (2013) identified them as supporting growth in satisfaction with life. Meanwhile, interest and joy have been described as related but rather distinct by Consedine et al. (2004). It could be of great interest to conduct additional studies, not only using questionnaires, such as the *PANAS*, as a general measure, but also to investigate the single predictive value of the included emotions in *path models*. Specifically, the above-mentioned clinical advances of Positive Psychology could greatly benefit from deeper insights into the interplay of positive emotions.

### Limitations and Differences

After finding justified evidence for a single factor model in emotions of Fredrickson (2013), we would like to point out several challenges and further perspectives. As mentioned before, the differences between the current study and the experiments by Hu et al. (2017, 2019) call the comparability of the results into question. While Hu et al. (2017, 2019) used an experimental setting, inducing emotions through stimuli and underpinning their findings with biological measurements in small sample sizes, we generated a large natural and non-manipulated sample. Also, we collected self-report data, taking the experience and remembered intensity of described emotions of the preceding days into account. This approach, while suitable for the collection of bigger samples, also triggers possible biases toward memory, regarding past emotions, which might, e.g., deviate from *reality*, influenced by the current feelings and situations that participants experienced during working on the survey.

This is also the subject of an ongoing debate concerning the accuracy of remembered emotions, as Thomas and Diener (1990), for example, found inconclusive results. Robinson and Clore (2002), as well as Levine et al. (2009) summarized possible biases and described criticism regarding emotional self-report about past events. This seems to account especially for complex and multidimensional emotions (Aaker et al., 2008) as *awe* (Chirico et al., 2017). As noted previously, our study in fact lacks biological measures, but is more representative for everyday life because of its focus, its broader age range, and its bigger sample size. Additionally, it could be interesting for future studies, to include intensity as well as frequency measures of emotional experience, to validate the proposed hierarchy of emotional experiences hypothesized by Fredrickson (2013) and investigate the comparability of factor structures in both frames.

## Conclusion

In sum, we showed that the latent factor structure of daily-life positive emotions apparently differs from experimentally elicited positive emotions. This seems trivial at first glance but presents serious questions with regards to experimental research on emotions and the external validity of its results. While emotion-elicitation in the lab seems valid, for example, if scientific interests rely on the observation of a specific emotion in a controlled setting, it seems questionable, however, if we are interested in the emotional experience and its dynamics in everyday life. This seems to offer a remarkable note to all research, which tries to derive “generalizable” results about emotional experience in laboratories, as artificially strong elicited emotions might show interrelations, as presented in Hu et al. (2017, 2019), while positive emotions in daily life feature the dynamics of a more general model, as presented in our paper.

In sum and as a general result concerning the state of research in this field, we come to the conclusion that valid knowledge about “theory” of the top 10 positive emotions of Frederickson (2013) is still preliminary. Therefore, research in this field is still in its infancy and thus, should get started at an international and intercultural level, before overgeneralizing singular results.

## Data Availability Statement

The raw data supporting the conclusions of this article will be made available by the authors, without undue reservation.

## Ethics Statement

Ethical review and approval was not required for the study on human participants in accordance with the local legislation and institutional requirements. The patients/participants provided their written informed consent to participate in this study.

## Author Contributions

LR collected and analyzed the data and wrote the draft. A-RL developed the structure of the text. A-RL and LR underwent several feedback loops. All authors contributed to the article and approved the submitted version.

### Conflict of Interest

The authors declare that the research was conducted in the absence of any commercial or financial relationships that could be construed as a potential conflict of interest.
