# Supplementary material for: Factor Structure of the “Top Ten” Positive Emotions of Barbara Fredrickson
Source: Front Psychol. 2021 May 14;12:641804. doi: 10.3389/fpsyg.2021.641804 (PMC8162787; doi:10.3389/fpsyg.2021.641804)
Supplement: Supplementary file 1 [file Data_Sheet_1.PDF]

Supplemental Material

Sampling and data extraction – Details

The study was designed, using soscisurvey.de, a commonly used platform for online questionnaire design and administration, which offers its services freely for non-commercial research interests. The design included, apart from the reported emotions and demographics, further scales for different purposes, which have been the LOT-R, RS-13, TST, scale for cognitive coping (Moos, 1984) and the SWLS. Yet, emotions have been measured first, to avoid biases through the following scales. Demographic questions have been tied to the end of the study. The link for the study was spread through social media, namely different facebook groups (mostly bound to students from the University of Vienna) and by personal accounts. Also, seminar participants have been encouraged to share the link with friends and relatives. Data extraction was handled with Excel for Mac. Only complete cases have been considered in the further progress of the study and therefore no specific handling of missing values is reported.

Descriptives

	% (*n*)		% (*n*)
Gender		Active Sexlife	
Female	62.2 (194)	*Yes*	58.3 (182)
Male	37.2 (116)	*No*	41.7 (130)
Other	0.6 (2)		
Education		Mothertongue	
Not university	7.7 (24)	*German*	87.8 (274)
Currently studying	47.1 (147)	*Other*	4.5 (14)
Finished studying	45.2 (141)	*German & other*	7.7 (24)
Income (subjective)		Mothertongue parents	
Not enough	15.4 (48)	*German*	83.0 (259)
Enough	60.9 (190)	*Other*	9.9 (31)
More than enough	23.7 (74)	*German & other*	7.1 (22)
Income (objective in Euro)		Religion	
0 – 500	11.5 (36)	*Christian*	41.3 (129)
501 – 1.000	27.6 (86)	*Jewish*	0.3 (1)
1.001 – 1.500	14.1 (44)	*Muslim*	1.3 (4)
1.501 – 2.000	11.9 (37)	*Atheist*	24.4 (76)
2.001 – 2.500	16.7 (52)	*Other*	4.8 (15)
> 2.500	18.3 (57)	*None*	27.9 (87)
Relationship		Housing	
Monogamous	57.7 (180)	*Alone*	18.9 (59)
Open relationship	2.6 (8)	*Flatshare*	34.3 (107)
Other concepts	4.5 (14)	*With family (e.g. Parents)*	12.8 (40)
Single	35.3 (110)	*With Partner*	30.1 (94)
		Other	3.8 (12)

Exact p-values of Correlation Table

	1	2	3	4	5	6	7	8	9	10
**Amusement**		0.091^{ns}	<.00001	<.00001	<.00001	<.00001	<.00001	<.00001	<.00001	<.00001
**Awe**			0.00028	0.01565	0.00011	<.00001	0.04215	0.03516	0.01454	0.0034
**Gratitude**				<.00001	<.00001	<.00001	<.00001	<.00001	<.00001	<.00001
**Hope**					<.00001	<.00001	<.00001	<.00001	<.00001	<.00001
**Interest**						<.00001	<.00001	<.00001	<.00001	<.00001
**Joy**							<.00001	<.00001	<.00001	<.00001
**Love**								<.00001	<.00001	0.00153
**Pride**									<.00001	<.00001
**Serenity**										<.00001
**Inspiration**										

List of Eigenvalues

ID	Eigenvalues
1	3.75
2	0.33
3	0.18
4	0.12
5	0.03
6	-0.03
7	-0.09
8	-0.14
9	-0.18
10	-0.23

Emotions – English and German

English	German
Amusement	Vergnügen
Awe	Ehrfurcht
Gratitude	Dankbarkeit
Hope	Hoffnung
Interest	Interesse
Joy	Freude
Love	Liebe
Pride	Stolz
Serenity	Heiterkeit
Inspiration	Inspiration
